# Supplementary material for: Identification of photoperiod-induced specific miRNAs in the adrenal glands of Sunite sheep (Ovis aries)
Source: Front Vet Sci. 2022 Jul 22;9:888207. doi: 10.3389/fvets.2022.888207 (PMC9354845; doi:10.3389/fvets.2022.888207)
Supplement: Supplementary file 1 [file Data_Sheet_1.ZIP › Supplementary file/miRDeep2 file.zip!/miRDeep2 file/Know_Structure/LP42A3/oar-mir-433.pdf]

miRBase precursor : oar-mir-433
Total read count : 2655
oar-miR-433-5p read count : 7
oar-miR-433-3p read count : 2648
remaining reads : 0

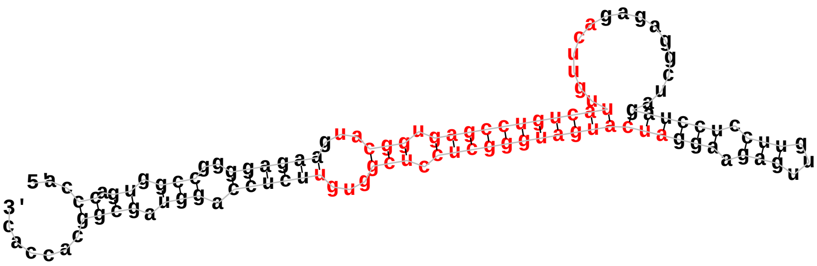

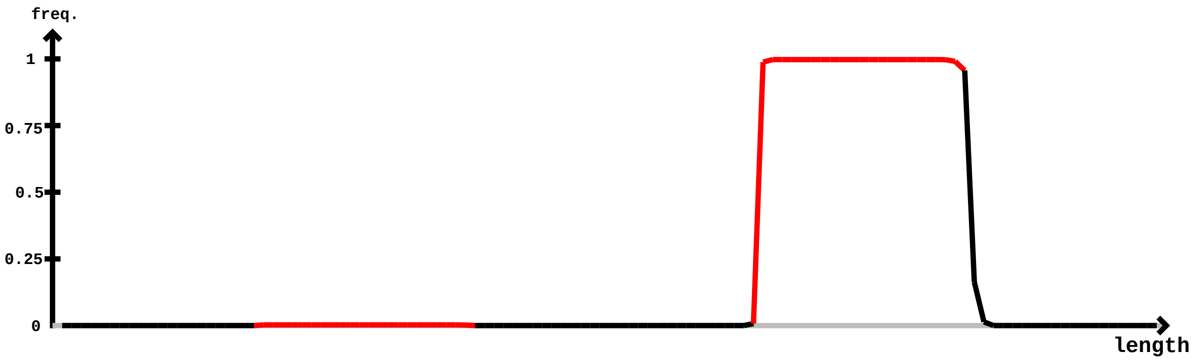

oar-miR-433-5p

oar-miR-433-3p

```
5'- acccaguggccggggagaaguacggugagccugucauuguucagagaggcuagauccuccuuguugagaaggaucaugaugggcuccucgguguucuccagguagcggcaccac   -3'    exp
    ..((.((.(((..((((((...(((.(((((((((((..............((((((.(((...))).))))))))))))))))).)))...)))))).))).))))......    reads      mm         sample
    ....................uacggugagccugucauuguu........................................................................    2          0          seq
    ....................uacggugagccugucauuguuc.......................................................................    5          0          seq
    ......................................................................gaucaugaugggcuccucggug.....................    1          0          seq
    ......................................................................gaucaugaugggcuccucggugu....................    18         0          seq
    ......................................................................gaucaugaugggcuccucgguguu...................    1          0          seq
    .......................................................................aucaugaugggcuccucgg.......................    1          0          seq
    .......................................................................aucaugaugggcuccucggu......................    17         0          seq
    .......................................................................aucaugaugggcuccucggug.....................    89         0          seq
    .......................................................................aucaugaugggcuccucggugu....................    2066       0          seq
    .......................................................................aucaugaugggcuccucgguguu...................    393        0          seq
    .......................................................................aucaugaugggcuccucgguguuc..................    37         0          seq
    ........................................................................ucaugaugggcuccucggugu....................    23         0          seq
    ........................................................................ucaugaugggcuccucgguguu...................    2          0          seq
```
